# Accelerated loop-mediated isothermal amplification method for the rapid detection of Streptococcus suis serotypes 2 and 14 based on single nucleotide polymorphisms

**DOI:** 10.3389/fcimb.2022.1034762

**Published:** 2022-11-11

**Authors:** Jiajia Meng, Chunling Li, Yu Wang, Zhibiao Bian, Pinpin Chu, Shaolun Zhai, Dongxia Yang, Shuai Song, Yan Li, Zhiyong Jiang, Kunli Zhang, Yugu Li, Hongchao Gou

**Affiliations:** ^1^ Guangdong Laboratory for Lingnan Modern Agriculture, College of Veterinary Medicine, South China Agricultural University, Guangzhou, China; ^2^ Institute of Animal Health, Guangdong Academy of Agricultural Sciences, Guangzhou, China; ^3^ Guangdong Provincial Key Laboratory of Livestock Disease Prevention, Guangzhou, China; ^4^ Maoming Branch, Guangdong Laboratory for Lingnan Modern Agriculture, Maoming, China; ^5^ Scientific Observation and Experiment Station of Veterinary Drugs and Diagnostic Techniques of Guangdong Province, Guangzhou, China

**Keywords:** *Streptococcus suis* serotypes 2 and 14, single-nucleotide polymorphism, rapid detection, RNase H2 enzyme, loop-mediated isothermal amplification

## Abstract

*Streptococcus suis* serotypes 2 and 14 are the most prevalent zoonotic strains. The establishment of a sensitive and extremely accurate method for point-of-care testing for *Streptococcus suis* serotype 2 and 14 strains is highly desirable. In this study, a loop primer probe-introduced loop-mediated isothermal amplification assay was developed to differentiate *Streptococcus suis* serotypes 2 and 14 based on SNP (single nucleotide polymorphism). The specific fluorescent probes were designed for the SNP site specific for serotype 2 and 14 *Streptococcus suis cps*K genes, and the loop primer probe-introduced loop-mediated isothermal amplification (LAMP) assay was developed using the specific cleavage properties of the RNase H2 enzyme. Rapid and efficient LAMP assays were realized through the use of loop forward primers and stem forward primers. The results showed that the amplification reaction can be performed efficiently at 59°C. The results can be real-time detected or judged using a smartphone and a 3D-printed visualization cassette. The sensitivity of the LAMP assay can reach 18.4 CFU within 40 minutes. The detection rate of the assay system was evaluated using 19 clinical samples with suspected *Streptococcus suis* infection, and the detection rate was consistent with the sequencing method, suggesting that the test is highly practical. The LAMP assay for *Streptococcus suis* serotypes 2 and 14 established in this study has strong specificity, high sensitivity, and simple operation, while the reaction can be performed at an isothermal temperature and is not dependent on complex instruments or professional operators, making it suitable for field testing.

## Introduction

*Streptococcus suis* is an encapsulated Gram-positive bacterium colonizing the respiratory and digestive tracts of pigs. To date, *Streptococcus suis* has been classified into 29 serotypes. Initially, *Streptococcus suis* was identified as having 35 serotypes (Liu et al., 2013). However, serotypes 20, 22, 26, 32, 33, and 34 were re-identified as other species (Gottschalk et al., 1989; Gottschalk et al., 1991; Higgins et al., 1995; Tien et al., 2013). *Streptococcus suis* is an important pathogen causing economic losses in the pig industry. Moreover, it is a zoonotic agent causing severe infections in people in close contact with infected pigs or pork-derived products (Goyette-Desjardins et al., 2014). Furthermore, *Streptococcus suis* serotypes 2 and 14 are the most prevalent zoonotic strains (Goyette-Desjardins et al., 2014). In the last several decades, the number of reported human cases of *Streptococcus suis* infection has rapidly increased, and three epidemics were recorded in China in 1998, 2005, and most recently in 2016 (Yang et al., 2006; Huang et al., 2019). As such, to achieve effective prevention and control of this disease, a rapid and accurate typing method is needed for the monitoring and analysis of zoonotic strains of *Streptococcus suis*, especially *Streptococcus suis* serotypes 2 and 14.

For the typing of *Streptococcus suis*, the traditional methods of bacterial isolation and biochemical analysis are time-consuming, laborious, and relatively insensitive. To date, several envelope polysaccharide-encoding gene (cps)-based multiplex PCR typing methods have been developed (Kerdsin et al., 2012; Liu et al., 2013; Kerdsin et al., 2014; Okura et al., 2014). A multiplex PCR method has been developed to detect *Streptococcus suis* serotype 1/2 and 2 or 1 and 14 (Kerdsin et al., 2014). However, because the *cps*K genes of these serotypes are highly homologous, these multiplex PCR-based assays do not distinguish serotype pairs 1/2 and 2 or 1 and 14 (Okura et al., 2014). It has been reported that a single nucleotide polymorphism (SNP) in the *cps*K gene of these serotypes at position 483 could differentiate between serotypes 1/2 and 2 as well as 1 and 14. *Streptococcus suis* serotype 2 and 14 strains have a G nucleotide at position 483 of the *cps*K gene, whereas serotype 1/2 and 1 strains contain either a C or T at this nucleotide position (Athey et al., 2016). Thus, PCR-restriction fragment length polymorphism (PCR-RFLP), rapid high resolution melting (HRM), and mismatch amplification mutation assays (MAMA-PCR) have been applied to differentiate among serotypes 1/2, 2, 1, and 14 (Lacouture et al., 2020; Matiasovic et al., 2020; Scherrer et al., 2020). However, these methods are complicated to operate and require special instruments, and thus establishing a sensitive and extremely accurate SNP detection method for point of care testing of *Streptococcus suis* serotype 2 and 14 strains on-site is highly desired.

The loop-mediated isothermal amplification (LAMP) method is a novel nucleic acid amplification technique that relies on four primers that recognize six specific regions on the target DNA and a DNA polymerase with strand-displacement activity to efficiently amplify nucleic acids at a constant temperature (Notomi et al., 2000). The method is characterized not only by its simplicity of operation, rapidity of reaction, and ease of detection but above all by the fact that it does not require a thermal cycler, and amplification can simply be carried out using a water bath or heating block that maintains the desired temperature (Wong et al., 2018). However, conventional LAMP has limited application for SNP detection (Srividya et al., 2019). In a previous study, we utilized the accurate cleavage ability of the RNase H2 enzyme on ribonucleotides and developed an accurate POCT (point of care testing) method for SNP detection (Wen et al., 2021). Here, we have developed a cleaved loop primer probe LAMP (LP-LAMP) method to realize POCT typing of *Streptococcus suis* serotypes 2 and 14 based on the SNP in the *cps*K locus. It has been demonstrated that this method has strong specificity, high sensitivity, and simple operation, while the reaction can be performed at isothermal temperatures, is not dependent on instruments or professional operators, and is suitable for field testing.

## Materials and methods

### Bacterial culture

A total of eight standard strains (serotype 1/2, serotype 5, serotype 7, serotype 23, serotype 28, serotype 29, serotype 31) and 19 clinical strains (**Table 1**) of *Streptococcus suis* with different serotypes are kept at the laboratory at Guangdong Animal Health Institute.

**Table 1:** Clinical *Streptococcus suis* strains.

NO.	*Streptococcus suis*	Multiplex PCR
1	GZ-156	serotype 2 or 1/2
2	GZ-894	serotype 2 or 1/2
3	GZ-1016	serotype 2 or 1/2
4	GZ-746	serotype 2 or 1/2
5	GZ-759	serotype 2 or 1/2
6	GZ-766	serotype 2 or 1/2
7	GZ-783	serotype 2 or 1/2
8	GZ-1018	serotype 2 or 1/2
9	GZ-804	serotype 2 or 1/2
10	GZ-820	serotype 2 or 1/2
11	GZ-834	serotype 2 or 1/2
12	GZ-840	serotype 2 or 1/2
13	GZ-905	serotype 2 or 1/2
14	GZ-953	serotype 2 or 1/2
15	GZ-964	serotype 2 or 1/2
16	GZ-975	serotype 2 or 1/2
17	GZ-1084	serotype 2 or 1/2
18	GZ-1079	serotype 2 or 1/2
19	GZ-1089	serotype 2 or 1/2

### Genomic DNA extraction

All of the bacterial strains were cultured in tryptone soy broth (TSB) plates at 37°C. The genomic DNA was extracted by the boiling method. In brief, 1 mL of bacterial solution was centrifuged at 12 000 r/min for 2 min. The supernatant was discarded. Then, 100 μL of PBS buffer was added to resuspend the pellet and centrifuged at 12 000 r/min for 4 min. This was repeated three times. After 100 μL of dd-H_2_O was added, the resuspension was boiled at 100°C for 5 min and centrifuged at 12 000 r/min for 2 min. The supernatant was used as a DNA amplification template and stored at −20°C.

### Primer design

LAMP primers were targeted to the *Streptococcus suis cps*K gene sequence (GenBank: AF118389) and designed by using PrimerExplorer V5 (http://primerexplorer.jp/e/). Probes were designed by using Primer Primer5 software to detect the 483rd position of the *cps*K gene. All primers and probes (**Table 2**) were synthesized by Sangon Biotech Co. Ltd. (Shanghai, China) and stored at –20°C until use.

**Table 2:** Primers of the LP-LAMP method for the *Streptococcus suis cps*K gene.

Primer name	Sequences 5’-3’	Genome position^a^
F3	GAGAGAATGCCCTTGTGG	143-160
B3	AGTATTCATCTTCATGAATCTTACC TCATAAGTAGAAGTCTTAGAATGG	352-376
FIP	GAACAGCCTGATTTGTAGGAAGC-GATAGGGTAGATGCTTCGCG	F1c+F2
BIP	GCGGATGGTCATCGCTTTGT-CTTTTCAAATCGAAAATCTTCA	B1c+B2
F2	GATAGGGTAGATGCTTCGG	175-193
F1c	GAACAGCCTGATTTGTAGGAAGC	216-238
B2	CTTTTCAAATCGAAAATCTTCA	330-351
B1c	GCGGATGGTCATCGCTTTGT	274-293
LF	GCTCTGCTGTTAAGAAATGC	195-214
SF	TACAAACATTCCTGCCGCTC	324-343
probe	TTTGTGGTGGCCT(FAM)GG(RNA)AAT(BHQ1)AAACTCT-C3 Spacer	

a The genome of Streptococcus suis cpsK gene (GenBank: AF118389).

### Basic LAMP reaction system

The reaction mixture of LP-LAMP consisted of 1.6 µM FIP (forward inner) primers and BIP (backward inner) primers, 0.2 µM F3 (outer forward) and B3 (outer backward) primers, 0.8 µM of LF (loop forward) primers, 0.4 µM probe, 8 U of Bst 2.0 DNA polymerase, 6 mM MgSO_4_, 1 × buffer (New England Biolabs Inc.), 5 mU of RNase H2 enzyme (catalog no. 11-02-12-01, Integrated DNA Technologies), and 1.6 µM of dNTPs (Vazyme). Then, 1 µL of the DNA sample was added, and the final volume was adjusted to 25 µL with dd-H_2_O. Before closing the lid, we applied mineral oil to the surface to prevent contamination. Each reaction mixture was incubated at 63°C for 60 minutes in a Roche Light Cycler 480 system.

### Optimization of the reaction system

For the LP-LAMP method, it is crucial to optimize the reaction temperature, the amount of the probe, and the RNase H2 enzyme. Therefore, the LAMP reaction temperature was optimized by setting a single-factor gradient, and the reaction temperature gradient was 58°C, 59°C, 60°C, 61°C, 62°C, 63°C, 64°C, 65°C, and 66°C using the TIANLONG Gentier 96R system. Then, the optimal probe dose was determined by performing an LP-LAMP test with dose series of 0 µM, 0.1 µM, 0.2 µM, 0.3 µM, 0.4 µM, 0.5 µM, and 0.6 µM. Similarly, the RNase H2 enzyme was set at different concentrations (0 mU, 1.25 mU, 2.25 mU, 2.5 mU, 5 mU,6.25 mU, and 7.5 mU) for LP-LAMP.

### Sensitivity test

*Streptococcus suis* serotype 2 was cultured at 37°C for 24 h. Ten-fold serial dilutions were performed with sterile distilled water, and the number of colony forming units (CFU) per mL was accurately determined by the plate count technique. Then, 1μL genomic DNA of each dilution was used as the template for amplification.

### Specificity test

The specificity of the LP-LAMP reaction was verified by using the genomic DNA from the other eight standard serotypes of *Streptococcus suis* (serotype 1/2, serotype 5, serotype 7, serotype 9, serotype 23, serotype 28, serotype 29, and serotype 31).

### Application of the LP-LAMP method in clinical detection

A total of 19 clinical bacterial strains were used to verify the analytical ability of the LP-LAMP assays. The 19 clinical strains were confirmed by using multiplex PCR, and the amplified products were subjected to agarose gel electrophoresis, showing 450 bp bands for types 1/2 and 2 and 550 bp bands for types 1 and 14 on the agarose gel (Kerdsin et al., 2014). At the same time, the bacterial broth was amplified by using the PCR method with F3/B3 primer and sent for sequencing. The sequencing results were compared with those of the LP-LAMP method.

## Results

### Principle of the LP-LAMP method

**Figure 1:**
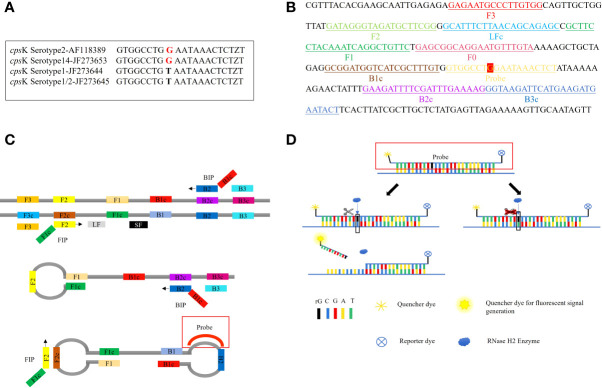
Principle of the LP-LAMP Method. **(A)** Single-nucleotide polymorphism at the 483rd site of the *cps*K gene for serotype 2, serotype 14, serotype 1, and serotype 1/2 *Streptococcus suis*. **(B)** Schematic diagram of the *cps*K gene showing the position and composition of LP-LAMP primers and probe. **(C)** The principle of the LP-LAMP method. **(D)** The working principle of the loop primer probe.

The 483rd position of the Serotype 2 and 14 *Streptococcus suis cps*K gene is a G, while for serotype 1 and 1/2 this is a T (**Figure 1A**). Hence, this site of the *cps*K gene is targeted to design an allelic discrimination loop primer probe with a ribonucleotide insertion based on the original LAMP primer sets (**Figure 1B**). The probe carries a reporter dye at the 5’ end and a quencher dye at the 3’ end. The intact probes show no fluorescence owing to the close proximity between the reporter dye and quencher dye (Kim and Misra, 2007). When the LAMP reaction occurs, the templates are amplified. Then, the loop primer binds to the corresponding region, and the ribonucleotide is exactly matched with the mutant site (**Figure 1C**). Furthermore, the hydrolytic mechanism of the RNase H2 enzyme is activated (Dobosy et al., 2011), cleaving the probe and separating the reporter dye from the quencher, resulting in the release of the fluorescent signal. In contrast, a mismatched probe remains intact and does not fluoresce (**Figure 1D**). Thus, robust and specific detection of SNPs is achieved (Shen et al., 2020).

### Establishment of the basic reaction system

**Figure 2:**
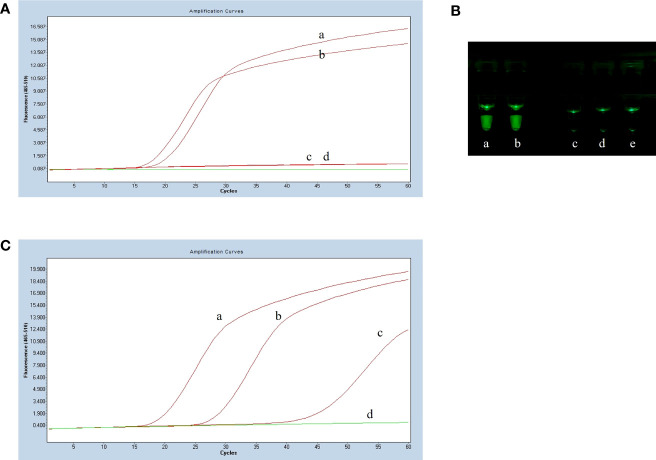
Results of establishing the basic reaction system. **(A)** Fluorescence amplification curve of the basic reaction. **(B)** Observing the basic reaction result with a 3D printed visual function box. (a) *Streptococcus suis* serotype 2 genomic DNA, (b) *Streptococcus suis* serotype 14 genomic DNA, (c), (d) and (e) Negative control. **(C)** The effect of the usage of the swarm primers. (a) LF primers and SF primers, (b) LF primers, (c) SF primers, (d) Negative control.

The LP-LAMP method was tested by using standard Serotype 2 and 14 *Streptococcus suis* strains (**Figure 2A**). Furthermore, to facilitate clinical testing a POCT platform was set up using a 3D-printed visualization cassette. After the LP-LAMP reaction, the tube containing the reaction mixture was placed in a 3D-printed visualization cassette. Using this device, which is available for smartphone photography and visualization, positives show green fluorescence, while negatives show no fluorescence (**Figure 2B**) (Wen et al., 2021). The results demonstrated that this novel SNP assay successfully detected *Streptococcus suis* serotypes 2 and 14. The basic LP-LAMP response was thus established. As was reported previously, the action of stem forward (SF) primers can be rationalized *via* the proposed mechanism of LAMP, which anneal to transiently single-stranded regions of the amplicon and recopy the entire binding sites for the BIP/FIP primers. An additional unique feature is the extra strong intra-molecular self-priming when stem forward primers delimit amplicons (Gandelman et al., 2011). To accelerate the LP-LAMP, SF primers were applied in this study. As shown in **Figure 2B**, the combination of LF primers and SF primers was the most effective compared with the LF primers alone or the SF primers alone. Therefore, the addition of SF primers was necessary for promoting the LP-LAMP (**Figure 2C**).

### Optimization results of basic reaction system

**Figure 3:**
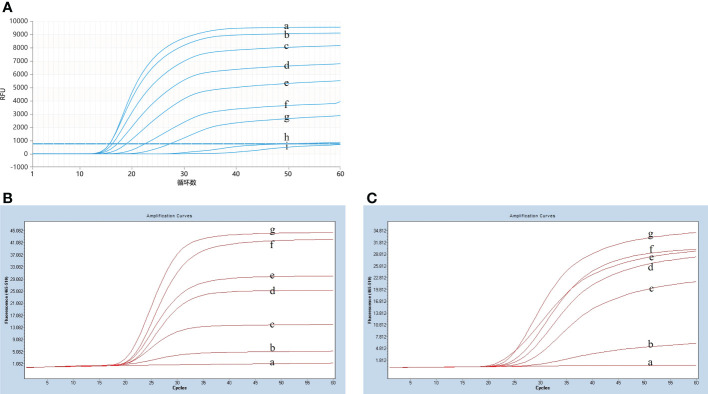
Results of reaction system optimization. **(A)** Optimization of temperature. (a) 58°C(b) 59°C, (c) 60°C, (d) 61°C, (e) 62°C, (f) 63°C, (g) 64°C, (h) 65°C, (i) 66°C. **(B)** Probe dosage optimization. (a) 0 µM, (b) 0.1 µM, (c) 0.2 µM, (d) 0.3 µM, (d) 0.4 µM, (e) 0.5 µM, (f) 0.6 µM, (g) 0.7 µM. **(C)** Optimization test for the RNase H2 enzyme. (a) 0 mU, (b) 1.25 mU, (c) 2.5 mU, (d) 3.75 mU, (e) 5 mU, (f) 6.25 mU, (g) 7.5 mU.

To optimize the reaction temperature for the LP-LAMP assay, the reaction mixtures were heated separately at 1°C intervals from 58°C to 66°C for 60 min. The results of the temperature optimization are shown in **Figure 3A**. The peak began earliest and the fluorescence value of the amplification curve was highest when the reaction temperature was 59°C. Therefore, the optimum temperature was designated as 59°C for the LP-LAMP. Then, the optimal probe reaction dose was determined by setting the probe dose of LP-LAMP from 0 µM to 0.7 µM (**Figure 3B**). When the probe concentration was 0.4 µM, there was good amplification efficiency and no major difference in dosage profiles compared to higher concentrations. The best reaction and amplification efficiency were achieved at a probe concentration of 0.4 µM, and therefore this was chosen as the optimum concentration. Likewise, an optimization test for the RNase H2 enzyme was carried out (**Figure 3C**). The results showed that an RNase H2 enzyme concentration of 5 mU had the best reaction efficiency and amplification efficiency. Therefore, 5 mU RNase H2 enzyme was set as the optimum dosage.

In summary, the optimal system for LP-LAMP consisted of 1.6 µM FIP and BIP primers, 0.2 µM F3 and B3 primers, 0.8 µM of LF and SF primers, 0.4 µM probe, 8 U of Bst 2.0 DNA polymerase, 6 µm MgSO_4_, 1 × buffer 2.5 mU of RNase H2 enzyme, 1.6 µM of dNTPs, and 1 µL of DNA sample. The optimal reaction temperature is 59°C.

### Sensitivity test results

**Figure 4:**
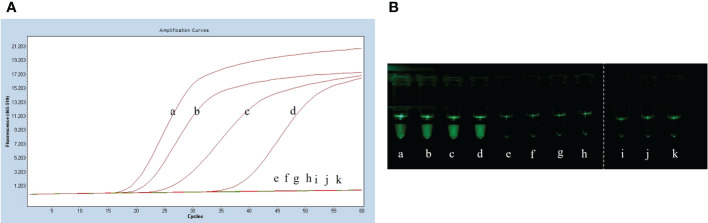
Sensitivity test results. **(A)** Sensitivity test real-time fluorescence curve results. **(B)** Visualization of the Sensitivity results using a printed visual function box. In the LP-LAMP method for the detection of genomic DNA, diluted *Streptococcus suis* serotype 2 genomic DNA was used at the following concentrations: (a) 1.84 × 10^4^ CFU, (b) 1.84 × 10^3^ CFU, (c) 1.84 × 10^2^ CFU, (d) 1.84 × 10^1^ CFU, (e) 1.84 × 10^0^ CFU, (f) 1.84 × 10^-1^ CFU, (g) 1.84 × 10^-2^ CFU, (h) 1.84 × 10^-3^ CFU, (i), (j) and (k) Negative control.

After plate counting of *Streptococcus suis* serotype 2 at a concentration of 1.84 × 10^5^ CFU, 1 µL of each sequential 10-fold gradient dilution was used as a template for LP-LAMP testing (**Figure 4A**). Visualization of the results of the specificity test (**Figure 4B**) showed that concentrations as low as 18.4 CFU were successfully amplified, indicating that the LP-LAMP test has good sensitivity.

### Specificity test results

**Figure 5:**
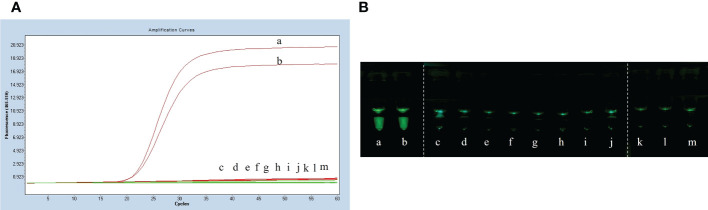
Specificity test results. **(A)** Specificity test real-time fluorescence curve results. **(B)** Visualization of the Specificity results by using a 3D printed visual function box. (a) *Streptococcus suis* serotype 2 genomic DNA, (b) *Streptococcus suis* serotype 14 genomic DNA, (c) serotype 1/2, (d) serotype 5,(e) serotype 7,(f) serotype 9, (g) serotype 23, (h) serotype 28, (i) serotype 29, (j) serotype 31,(k), (l) and (m) Negative control.

The specificity assay of LP-LAMP was performed using *Streptococcus suis* genomic DNA of eight standard strains (serotype 1/2, serotype 5, serotype 7, serotype 9, serotype 23, serotype 28, serotype 29, and serotype 31). The results are shown in **Figure 5A**, where serotypes 2 and 14 *Streptococcus suis* genomic DNA produced an amplification curve, while the other strains did not. The specificity test using self-designed 3D-printed dual-function visualization cassettes yielded consistent results (**Figure 5B**). Thus, the LP-LAMP method appeared to be highly specific.

### Application results of LP-LAMP method

**Figure 6:**
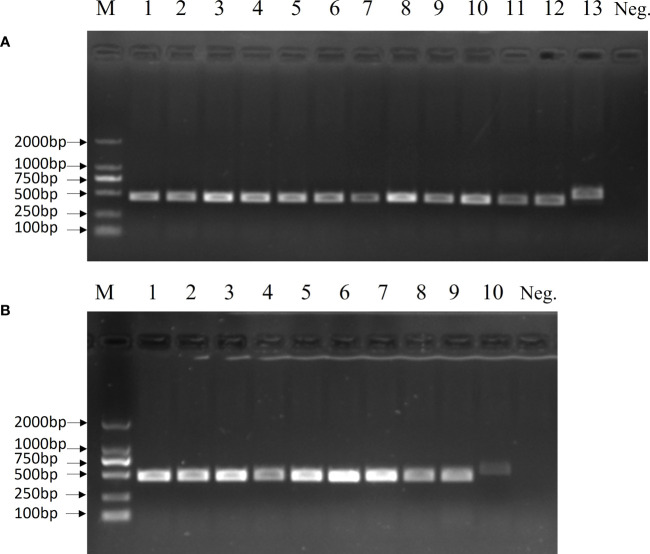
Multiplex PCR results for 19 clinical samples. **(A)** Lane M, DL-2000 DNA marker; Lanes 1–11: Clinical strains; Lane 12: *Streptococcus suis* serotype 2; Lane 13, *Streptococcus suis* serotype 14; Neg., Negative control. **(B)** Lane M, DL-2000 DNA marker; Lanes 1–8: Clinical strains; Lane 9: *Streptococcus suis* serotype 2; Lane 10: *Streptococcus suis* serotype 14; Neg., Negative control.

The 19 clinical strains were confirmed by using the multiplex PCR method, and the results are shown as 450 bp bands on agarose gels (**Figure 6**). These strains were sent for sequencing, and the results showed that 16 strains had G nucleotides at position 483 of the *cps*K gene and three strains had T. This means that 16 of these strains were serotype 2 *Streptococcus suis* and three were 1/2 *Streptococcus suis*. The DNA from the 19 clinical strains of *Streptococcus suis* was used to test the LP-LAMP method. The LP-LAMP result showed amplification curves in 16 strains and no amplification curves in three strains. By comparison, the results of the LP-LAMP method and the sequencing method were consistent (**Table 3**).

**Table 3:** The application of the LP-LAMP method in POCT of clinical *Streptococcus suis* strains.

NO.	Sequencing results for locus *cps*K 483	LP-LAMP	Multiplex PCR
1	G	+	serotype 2 or 1/2
2	T	−	serotype 2 or 1/2
3	G	+	serotype 2 or 1/2
4	G	+	serotype 2 or 1/2
5	G	+	serotype 2 or 1/2
6	G	+	serotype 2 or 1/2
7	G	+	serotype 2 or 1/2
8	T	−	serotype 2 or 1/2
9	G	+	serotype 2 or 1/2
10	G	+	serotype 2 or 1/2
11	G	+	serotype 2 or 1/2
12	G	+	serotype 2 or 1/2
13	G	+	serotype 2 or 1/2
14	G	+	serotype 2 or 1/2
15	G	+	serotype 2 or 1/2
16	T	−	serotype 2 or 1/2
17	G	+	serotype 2 or 1/2
18	G	+	serotype 2 or 1/2
19	G	+	serotype 2 or 1/2

(+) Positive, (−) Negative.

## Discussion

*Streptococcus suis* is considered a leading infectious disease in the pig industry and is clinically characterized by meningitis, septicemia, or arthritis, resulting in significant economic losses worldwide each year. *Streptococcus suis* has been reported to cause human infections, and most of the strains causing human infections are serotype 2 or 14. It is difficult to differentiate between serotype 1/2 and serotype 2 or between serotype 1 and serotype 14 of *Streptococcus suis*. The zoonotic potential of serotypes 2 and 14 emphasizes the need to accurately differentiate between serotypes 1 and 14 and serotypes 1/2 and 2 (Smith et al., 1999). Thus, serotyping of *Streptococcus suis* 2 and 14 strains is essential for infection control efforts and for reducing the likelihood of zoonotic outbreaks.

**Figure 7:**
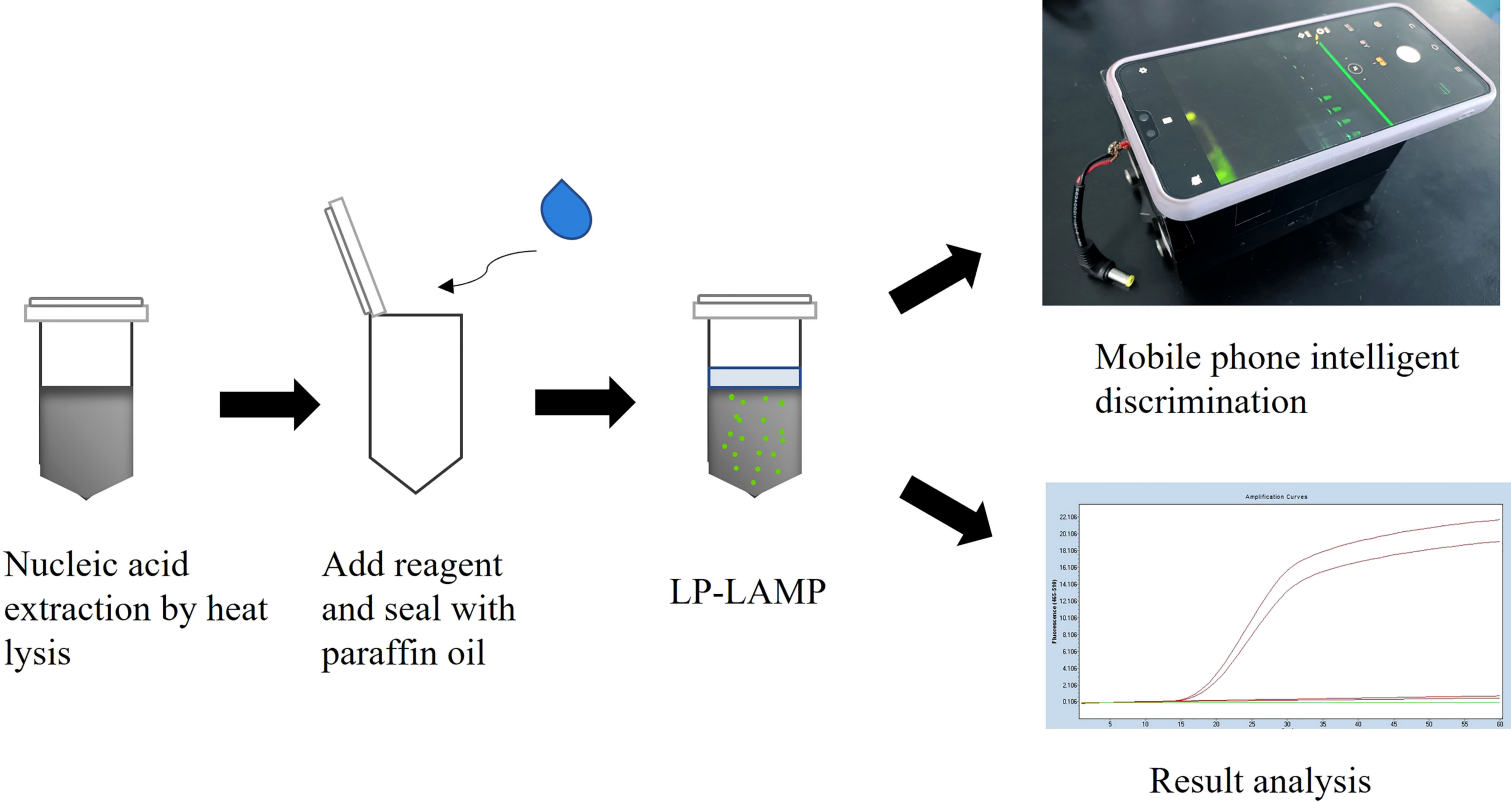
General workflow for the detection of *Streptococcus suis* serotype 2 or 14 strains by using LP-LAMP.

In this study, a one-loop primer LAMP typing assay was established using the serotype 2 and 14 *Streptococcus suis cps*K genes as target genes. By coupling with a multiplex PCR typing method, the test accurately distinguishes between serotype 2 and 14 *Streptococcus suis*. In this method, we designed a loop primer probe with ribonucleotide insertion through an SNP site of *Streptococcus suis* serotype 2 and serotype 14, different from serotype 1/2 and serotype 1. The SNP site is cleaved by the RNase H2 enzyme only when the base sequence exactly matches the base sequence of the mutation target to excite a fluorescent signal. By this design, the specificity of the probe is greatly improved, and the SNP can be unambiguously identified. In addition to real-time detection, the LP-LAMP method also uses a self-designed 3D-printed dual-function cassette for visualization and easy access to results (**Figure 7**). Thus, our simple procedure meets the needs of POCT and has the potential for clinical application in the field.

The LP-LAMP protocol developed has a detection limit of up to 18.4 CFU. The method can be completed at the limit of detection in 40 min. By adding LF primers and SF primers, the amplification efficiency is improved compared to the normal LAMP, thus obtaining the assay results in a shorter time. The LP-LAMP protocol has the potential to be well-aligned with sequencing methods. Compared with RFLP-based SNP detection techniques, our method eliminates the need for cumbersome and time-consuming follow-up procedures and the need to open tubes for electrophoresis compared to previously reported assays such as PCR-RFLP or MAMA-PCR. The entire assay process can be completed under closed-tube conditions, thus avoiding aerosol contamination. In contrast to HRM, the LP-LAMP method is not dependent on specialist instruments and does not require precise temperature control as long as the reaction is carried out at a constant temperature of 59°C (this can be achieved using a water bath on a metal block). Moreover, the LP-LAMP protocol time was reduced by 50 minutes compared to the 90-minute assay time of the HRM method. This makes for user-friendly POCT detection and reduces the cost. When 19 clinical samples were tested and analyzed by sequencing, the LP-LAMP results were consistent with the sequencing results, indicating high accuracy.

In conclusion, the present study developed a loop-mediated isothermal amplification method for the rapid detection of *Streptococcus suis* serotypes 2 and 14 based on a single nucleotide polymorphism. The method was low cost and simple to operate; it had a short detection time, high sensitivity, high specificity, and low requirements for basic laboratory facilities, in addition to being suitable for rapid diagnosis at the grassroots level and in the field. These properties indicated that the test could have good application prospects.

## Data availability statement

The raw data supporting the conclusions of this article will be made available by the authors, without undue reservation.

## Author contributions

JM carried out the experiment design and drafted the manuscript. YW, ZB, PC, and DY participated in the experiments. SZ, SS, ZJ, YL, and KZ participated in the analysis of the data. CL, YG-L, and HG conceived the study. All authors have read and approved the final manuscript.

## Funding

This work was supported by grants from the Special fund for scientific innovation strategy-construction of high level Academy of Agriculture Science-Distinguished Scholar (R2020PY-JC001), the Project of Collaborative Innovation Center of GDAAS (XTXM202202), the Project of Enterprise Science and Technology Commissioner of Guangdong Province (GDKTP2021018900), the Project of Agricultural Monitoring and Testing Project of Guangdong Province, the Project of Modern Agricultural Technology System Innovation Team of Guangdong Province (2021KJ119), the Guangzhou Science and Technology Correspondent Project (202121OO027), the Scientific innovation strategy-construction of high level Academy of Agriculture Science (202110TD, 202122TD, R2017YJ-YB2005 and R2021PY-QY006), and the Start-up Research Project of Maoming Laboratory (2021TDQD002).

## Conflict of interest

The authors declare that the research was conducted in the absence of any commercial or financial relationships that could be construed as a potential conflict of interest.

## Publisher’s note

All claims expressed in this article are solely those of the authors and do not necessarily represent those of their affiliated organizations, or those of the publisher, the editors and the reviewers. Any product that may be evaluated in this article, or claim that may be made by its manufacturer, is not guaranteed or endorsed by the publisher.
